# Characteristics of Fundus Autofluorescence in Active Polypoidal Choroidal Vasculopathy

**DOI:** 10.4274/tjo.54280

**Published:** 2016-08-15

**Authors:** Zafer Öztaş, Jale Menteş, Serhad Nalçacı, Mine Barış

**Affiliations:** 1 Ege University Faculty of Medicine, Department of Ophthalmology, İzmir, Turkey; 2 Buca Seyfi Demirsoy State Hospital, Ophthalmology Clinic, İzmir, Turkey

**Keywords:** Autofluorescence, fundus autofluorescence, polypoid, Polypoidal choroidal vasculopathy, branching vascular network

## Abstract

**Objectives:**

To define characteristic fundus autofluorescence (FAF) findings in eyes with active polypoidal choroidal vasculopathy (PCV).

**Materials and Methods:**

Thirty-five eyes of 29 patients with active PCV who were diagnosed at Ege University Faculty of Medicine, Department of Ophthalmology, Retina Division between January 2012 and November 2014 were included in the study. All the patients underwent a complete ophthalmological examination including fundus photography, spectral-domain optical coherence tomography, fluorescein angiography, FAF photography, and indocyanine green angiography (ICGA). ICGA was used to diagnose active PCV and identify lesion components. FAF findings were described at the retinal site of the corresponding lesions identified and diagnosed using ICGA.

**Results:**

The mean age of the 29 study patients (15 men, 14 women) was 64.6±7.5 years (range, 54-82 years). ICGA revealed active PCV in 35 eyes, consisting of polypoid lesions in 11 eyes (31.4%), branching vascular networks (BVN) in 10 eyes (28.6%), and a combination of polypoid lesions and BVNs in 14 eyes (40%). On FAF images, 4 different patterns were detected at the corresponding retinal sites of 25 polypoid lesions detected by ICGA: confluent hypoautofluorescence with a hyperautofluorescent ring in 18 eyes (72%), hyperautofluorescence with hypoautofluorescent ring in 2 eyes (8%), confluent hypoautofluorescence in 1 eye (4%), and granular hypoautofluorescence in 1 eye (4%). The remaining 3 eyes (12%) demonstrated blocked hypoautofluorescence because of the excessive hemorrhaging in the macula. The FAF images showed the granular hypoautofluorescent FAF pattern in all 24 BVNs (100%) consistent with the location of the lesions on ICGA.

**Conclusion:**

The typical PCV lesions, polypoid lesions and BVNs had characteristic autofluorescent findings on FAF imaging. Non-invasive, quick, and repeatable FAF imaging can be considered a reliable and helpful diagnostic technique for the diagnosis of active PCV.

## INTRODUCTION

Polypoidal choroidal vasculopathy (PCV) commonly manifests with serous and hemorrhagic retinal pigment epithelium (RPE) detachment and is characterized by polypoidal vascular dilations and/or abnormal branching vascular networks (BVN) in the inner choroidal vessels.^1^ First described by Yanuzzi et al.^1^ in 1982, the debate continues to this day whether PCV is a separate entity or a subtype of neovascular age-related macular degeneration (nv-AMD). The natural course and treatment of PCV are distinct from nv-AMD, which is clinically significant for differential diagnosis.^2,3,4^

Indocyanine green angiography (ICGA) imaging is accepted as the gold standard diagnostic method for PCV due to its ability to visualize lesion components like polypoidal dilations, BVN and choroidal vascular hyperpermeability (CVH). However, several challenges to the clinical application of ICGA are the long time required for imaging, difficulty in obtaining the dye, and equipment requirements. There is therefore a need for other PCV diagnostic methods that are less invasive, require less time and are easily repeatable.

Fundus autofluorescence (FAF) imaging is a diagnostic method which, instead of using fluorescent dye, is based on the inherent fluorescence of ocular structures. When stimulated with light of specific wavelengths, these structures autofluoresce at longer wavelengths due to the presence of fluorophore molecules. This autofluorescence (AF), which comes from N-retinyliden-N-retinylethanolamin (A2-E) found in lipofuscin granules in the retinal pigment epithelium, can be easily visualized using a fundus camera or a confocal scanning laser microscope modified with AF filters. FAF imaging provides valuable information concerning the metabolism and function of the RPE, and is a noninvasive, rapid, reliable and repeatable diagnostic method that has utility in the diagnosis of many diseases affecting the outer retinal layers.^5,6^

In this clinical study we aimed to determine the characteristic FAF appearance of lesion components like polypoidal structures and BVNs in eyes with active PCV using short-wavelength confocal scanning laser ophthalmoscopy (cSLO).

## MATERIALS AND METHODS

This prospective study included 35 eyes of 29 patients (15 male, 14 female) who were referred to the Ege University Faculty of Medicine, Department of Ophthalmology, Retina Unit with an initial diagnosis of nv-AMD and were diagnosed with active PCV between January 2012 and November 2014. All patients underwent a comprehensive ophthalmologic examination including medical history, best corrected visual acuity (BCVA), slit-lamp examination, fundus photography, spectral domain optical coherence tomography (SD-OCT), fluorescein angiography, FAF imaging and ICGA. SD-OCT images were acquired with the Heidelberg Spectralis HRA-OCT device (Heidelberg Engineering, Heidenberg, Germany) which operates with the cSLO technique. During the same session, ICGA was performed, OCT images were acquired at the retina sites corresponding to lesions found on ICGA, and FAF images were recorded using using 488 nm wavelength argon blue laser excitation with a 500 nm barrier filter.

The mean age of the patients was 64.6±7.5 (range, 54-82) years and mean BCVA was 0.28±0.28 Snellen (range, light perception-1.0).

PCV diagnosis was based on the detection of hyperfluorescent polypoidal focal choroidal vascular dilations and/or BVN lesions on ICGA, especially within the first 6 minutes. PCV was considered active in the presence of subretinal or intraretinal fluid, hemorrhage, pigment epithelium detachment or signs of leakage on FAF.

FAF images of the areas corresponding to polypoid lesions or BVNs on ICGA were described in terms of hyperautofluorescence and hypoautofluorescence patterns. The terms hyper- and hypoautofluorescence refer to areas of increased or decreased AF compared to the normal fundus. Hypoautofluorescent signals were further subgrouped as ‘confluent hypoautofluorescence’ and ‘granular hypoautofluorescence’. Confluent hypoautofluorescence describes areas of homogenously decreased AF which can be easily distinguished from the surrounding fundus; granular hypoautofluorescence describes heterogenous areas containing lesions with various degrees of hypoautofluorescence. In addition to these terms, cases in which hemorrhagic lesions impeded the normal autofluorescent signal of the fundus were described as ‘blocked AF’.

## RESULTS

**Figure 1:**
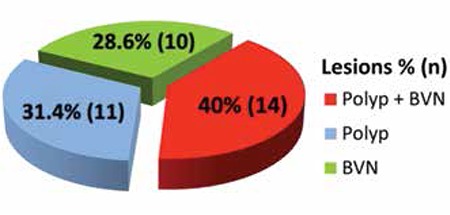
Distribution of polypoidal choroidal vasculopathy lesion components visualized by indocyanine green angiography BVN: branching vascular networks

Of the 35 eyes diagnosed with active PCV, ICGA revealed polypoid lesions only in 11 eyes (31.4%), BVNs only in 10 eyes (28.6%), and both polypoid lesions and BVNs in 14 eyes (40%) (Figure 1).

**Figure 2:**
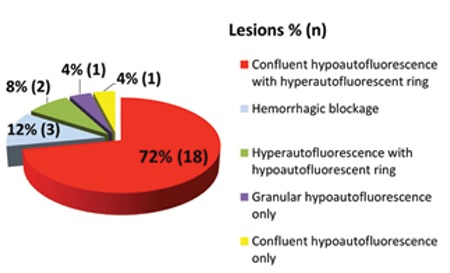
Fundus autofluorescence patterns and distribution of retinal areas corresponding to polypoid lesions on indocyanine green angiography

**Figure 3:**
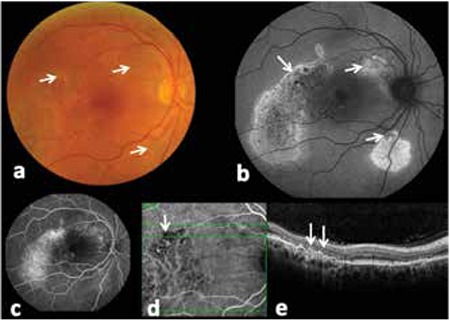
Areas of branching vascular networks (white arrows) in the right eye of a 72-year-old male patient visualized by a) color fundus photography, b) fundus autofluorescence, c) fluorescein angiography, d) indocyanine green angiography and e) spectral domain optical coherence tomography. The granular hypoautofluorescent fields on fundus autofluorescence represent the areas of branching vascular networks determined on indocyanine green angiography and spectral domain optical coherence tomography

Four different patterns were found in the FAF images of retinal areas corresponding to the 25 polypoid lesions detected by ICGA. These patterns were: confluent hypoautofluorescence with surrounding hyperautofluorescent ring in 72% (n=18), hyperautofluorescence with surrounding hypoautofluorescent ring in 8% (n=2), confluent hypoautofluorescence in 4% (n=1), and granular hypoautofluorescence in 4% (n=1). Blocked hypoautofluorescence was observed in 12% (n=3) of the eyes due to hemorrhage (Figure 2). All of the 24 BVN lesions (100%) showed granular hypoautofluorescence on FOF in the areas corresponding to the lesion on ICGA (Figure 3).

**Figure 4:**
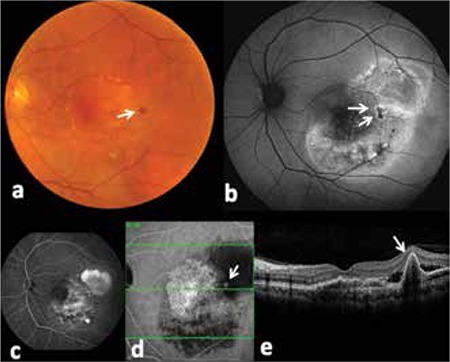
Images of a large polyp (white arrows) in the left eye of a 62-year-old male patient acquired by a) color fundus photography, b) fundus autofluorescence, c) fluorescein angiography, d) indocyanine green angiography and e) spectral domain optical coherence tomography imaging. A lesion presenting as subretinal hemorrhage on color fundus photography was determined to be an active polyp on indocyanine green angiography and spectral domain optical coherence tomography. The lesion exhibited confluent hypoautofluorescence with surrounding hyperautofluorescent ring on fundus autofluorescence imaging

SD-OCT sections obtained at areas exhibiting the most common polypoid lesion pattern of confluent hypoautofluorescence with surrounding hyperfluorescent ring revealed typical OCT appearance of polypoid lesions forming spindly processes on the posterior RPE surface with moderate interior reflectivity (Figure 4). Merging the OCT and FAF images showed that the areas of confluent hypoautofluorescence with surrounding hyperfluorescent ring on FAF corresponded to the sharp protusions of RPE detachment due to polypoid lesions.

## DISCUSSION

The eyes with active PCV in this study exhibited FAF patterns characteristic of polypoid lesions and BVNs. In FAF imaging, the conventional short-wavelength (488 nm) cSLO method was used in order to visualize the natural fluorescence arising due to the distribution of lipofuscin in the RPE.

Four distinct FAF patterns were observed in the polypoid lesions in the current study. The most common pattern was confluent hypoautofluorescence with hyperautofluorescent halo (72%), followed by the opposite pattern, hyperautofluorescence with hypoautofluorescent ring (8%). All of the BVN lesions (100%) showed granular hypoautofluorescence on FAF.

There are a few clinical studies in the literature which investigated the FAF characteristics of PCV.^7,8,9^

Yamagishi et al.^7^ analyzed the FAF features of polypoid lesions and BVNs in eyes with PCV. They observed two typical FAF patterns, reporting that all polypoid lesions exhibited central confluent hypoautofluorescence and the majority (80.4%) had a circumferential hyperautofluorescent ring. The second FAF pattern they observed was granular hypoautofluorescence in 98.9% of the areas correspondent to BVNs. In another study, Yamagishi et al.^9^ detected the polypoid lesion FAF pattern of confluent hypoautofluorescence with hyperautofluorescent ring in only 74% of patients.

Suzuki et al.^8^ described the FAF features of polypoid lesions in eyes with PCV and their changes after 3 years’ follow-up. They found that initially only 94.4% of polypoid lesions showed abnormal appearance on FAF, with the most common pattern (86.1%) being central hypoautofluorescence with a hyperautofluorescent ring. They also reported an abnormal FAF appearance (granular hypoautofluorescence) in only 67.7% of BVN lesions. The initial FAF appearance of the remaining 5.6% of polypoid lesions and 22.9% of BVN lesions was normal; however, by the end of the 3-year follow-up period there was a significant decrease in the BVNs with normal FAF appearance (6.2%).

The FAF patterns of polypoid lesions and BVNs most commonly observed in the current study were totally consistent with those reported in other studies. In addition, two other distinct FAF patterns were observed for polypoid lesions and the term ‘blocked hypoautofluorescence’ due to hemorrhage was used for the first time. Furthermore, none of the patients in our study exhibited normal FAF appearance. We believe this discrepancy between studies may be due to the fact that all of the patients included in our study had active and symptomatic manifestations of PCV. It seems that in other studies, PCV patients were not separated based on clinical characteristics like disease activity or the presence of symptoms.

The central hypoautofluorescence shown by polypoid lesions on FAF has been attributed to the formation of anterior processes in the RPE layers overlying the lesions; it has been claimed that these anatomic changes, which can also be detected on OCT, may lead to cellular damage by creating mechanical stress and pressure in the RPE layers.^9^ In fact, in many studies the polypoid structures and BVN typical of PCV have been described as lesions found just beneath the RPE which alter its morphology and in time even cause structural changes in the RPE. Analyses using OCT have revealed the close relationship between PCV lesions and the RPE. Histopathologic studies have demonstrated disruptions in the continuity of the RPE in PCV patients and proposed that this may arise due to abnormal hemodynamics in the choroidal vessels.^10^ The structural changes in the RPE caused by PCV lesions affects the distribution of lipofuscin. This increases the probability of being reflected on FAF imaging, which provides important information about the metabolism and function of the RPE, and creating the characteristic FAF appearance of lesions.

Central hypoautofluorescence is a common FAF pattern for polypoid lesions in all studies, which is considered indicative of permanent RPE atrophy and damage.^11^

## CONCLUSION

The results of our study support that FAF imaging is a noninvasive, rapid, repeatable diagnostic method that is useful in supporting the diagnosis of active PCV by ICG and OCT.

### Ethics

Ethics Committee Approval: It was taken, Informed Consent: It was taken.

Peer-review: Externally peer-reviewed.
